# Postoperative antibiotic prophylaxis for percutaneous nephrolithotomy and risk of infection: a systematic review and meta-analysis

**DOI:** 10.1590/S1677-5538.IBJU.2023.0626

**Published:** 2024-03-18

**Authors:** Thalita Bento Talizin, Alexandre Danilovic, Fabio Cesar Miranda Torricelli, Giovanni S. Marchini, Carlos Batagello, Fabio C. Vicentini, William Carlos Nahas, Eduardo Mazzucchi

**Affiliations:** 1 Universidade de São Paulo Hospital das Clínicas São Paulo SP Brasil Hospital das Clínicas, Universidade de São Paulo - USP, São Paulo, SP, Brasil;; 2 Hospital Alemão Oswaldo Cruz São Paulo SP Brasil Hospital Alemão Oswaldo Cruz, São Paulo, SP, Brasil;; 3 Universidade de São Paul Faculdade de Medicina Disciplina de Urologia São Paulo SP Brasil Disciplina de Urologia, Faculdade de Medicina, Universidade de São Paulo - USP, São Paulo, SP, Brasil;; 4 Instituto do Câncer do Estado de São Paulo Divisão de Urologia São Paulo SP Brasil Divisão de Urologia, Instituto do Câncer do Estado de São Paulo - ICESP, São Paulo, SP, Brasil

**Keywords:** Prostatic Neoplasms, Robotic Surgical Procedures, Diagnosis

## Abstract

**Purpose:**

The aim of this study is to perform a high-quality meta-analysis using only randomized controlled trials (RCT) to better define the role of postoperative antibiotics in patients undergoing percutaneous nephrolithotomy (PCNL).

**Materials and Methods:**

A literature search for RCTs in EMBASE, PubMed, and Web of Science up to May 2023 was conducted following the PICO framework: Population—adult patients who underwent PCNL; Intervention—postoperative antibiotic prophylaxis until nephrostomy tube withdrawal; Control—single dose of antibiotic during the induction of anesthesia; and Outcome—systemic inflammatory response syndrome (SIRS) or sepsis and fever after PCNL. The protocol was registered on the PROSPERO database (CRD42022361579). We calculated odds ratios (OR) and 95% confidence intervals (CI). A random-effects model was employed, and the alpha risk was defined as < 0.05.

**Results:**

Seven articles, encompassing a total of 629 patients, were included in the analysis. The outcome of SIRS or sepsis was extracted from six of the included studies, while the outcome of postoperative fever was extracted from four studies. The analysis revealed no statistical association between the use of postoperative antibiotic prophylaxis until nephrostomy tube withdrawal and the occurrence of SIRS/sepsis (OR 1.236, 95% CI 0.731 – 2.089, p=0.429) or fever (OR 2.049, 95% CI 0.790 – 5.316, p=0.140).

**Conclusion:**

Our findings suggest that there is no benefit associated with the use of postoperative antibiotic prophylaxis until nephrostomy tube withdrawal in patients undergoing percutaneous nephrolithotomy (PCNL). We recommend that antibiotic prophylaxis should be administered only until the induction of anesthesia in PCNL.

## INTRODUCTION

Currently, percutaneous nephrolithotomy (PCNL) stands as the gold standard treatment for kidney stones larger than 20 mm (1). However, infectious complications in PCNL pose a significant life-threatening concern. Fever is estimated to occur in up to 18% of patients, systemic inflammatory response syndrome (SIRS) in up to 35%, and sepsis in up to 6% (2-5). The use of antimicrobials in the perioperative period is a topic of ongoing discussion among specialists. There is currently no consensus regarding the optimal regimen and timing for administering antibiotics to these patients (1).

Although there is currently no evidence supporting the benefit of prophylaxis extended beyond 24 hours or until the removal of catheters, it is noteworthy that many urologists continue to use postoperative antibiotics until nephrostomy tube withdrawal (6-8). Urological guidelines explicitly state that there is no added benefit beyond single-dose prophylaxis (1). However, this statement is grounded in limited randomized controlled trials (RCTs). Our hypothesis posits that a meta-analysis could contribute a higher level of evidence on this subject, aiding urologists in adopting the best available practices. Presently, there is no meta-analysis that selects articles with a substantial number of patients to provide evidence against the prescription of antimicrobials until tube and nephrostomy removal. The practice of antibiotic maintenance remains prevalent in prescriptions worldwide. The objective of this study is to conduct a high-quality meta-analysis utilizing only RCTs to define the role of postoperative antibiotics in patients undergoing PCNL.

## MATERIALS AND METHODS

### Identification and Eligibility of Trials

This review adhered to the Preferred Reporting Items for Systematic Reviews and Meta-Analyses (PRISMA) statement (9). We exclusively included RCTs that compared a single dose of antibiotic during the induction of anesthesia with postoperative antibiotic prophylaxis until nephrostomy tube withdrawal in patients undergoing PCNL. Articles were retrieved from the EMBASE, PubMed, and Web of Science databases up to May 2023. Exclusion criteria encompassed observational and retrospective studies, case reports, case-control studies, letters to the editor, editorials, congress abstracts, and studies involving patients under 18 years old. The meta-analysis protocol was duly registered on the PROSPERO database on October 1, 2022 (CRD42022361579).

### Development of Prospective Meta-analysis Protocol

The PICO (Population, Intervention, Control, and Outcome) framework was established prior to data collection, as follows:

– Population: Adult patients (> 18 years old) who underwent PCNL.
– Intervention: Postoperative antibiotic prophylaxis until nephrostomy tube withdrawal.
– Control: Single dose of antibiotic administered during the induction of anesthesia.
– Outcome: SIRS or sepsis, and fever after PCNL.

### Outcomes and Comparisons

The primary outcome measure for this study was SIRS or sepsis after PCNL, with a comparison between a single dose of antibiotic during the induction of anesthesia and postoperative antibiotic prophylaxis until nephrostomy tube withdrawal. The secondary outcome was the occurrence of fever after PCNL. The definition of SIRS or sepsis was based on the criteria specified in each individual study (10, 11).

### Assessment of risk of bias in included studies

The assessment of risk of bias was conducted independently by two investigators, and any discrepancies were resolved through agreement. The risk of bias for each RCT was evaluated using version 2 of the Cochrane Risk of Bias Assessment Tool (RoB 2). RoB 2 is organized into domains of bias, encompassing trial design, conduct, and reporting of results, and is classified as unclear, low, or high risk (12).

### Data Analyses

All analyses were conducted using MedCalc for Windows, version 19.4 (MedCalc Software, Ostend, Belgium). The primary outcome was extracted from six out of the seven included studies, while the secondary outcome was extracted from four out of the seven included studies. Odds ratios (OR) and 95% confidence intervals (CI) were calculated for each study to assess differences among them. Heterogeneity was evaluated using the Chi-squared test and I2. A random-effects model was applied. The alpha risk was defined as < 0.05.

## RESULTS

### Search results and selection process

**Figure 1:**
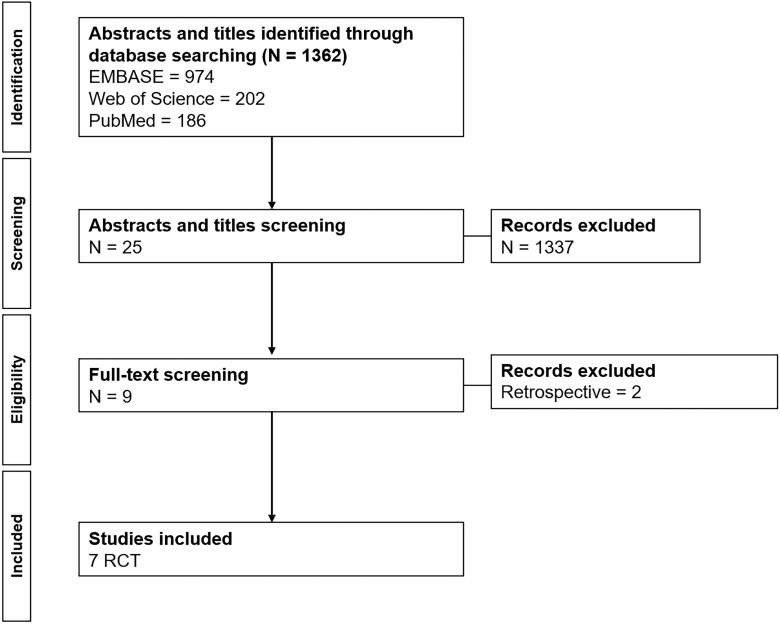
PRISMA flowchart.

In May 2023, a search strategy for "percutaneous nephrolithotomy" and "antibiotic" was executed on EMBASE (974 results), PubMed (197 results), and Web of Science (202 results) platforms, yielding a total of 1362 publications, as illustrated in Figure-1. Screening of abstracts and titles was performed, resulting in the exclusion of all studies that were not RCTs. Following full-text screening, seven RCTs were ultimately chosen, and two retrospective studies were excluded from the final selection. Consequently, the final set comprised seven RCTs, involving a total of 629 patients.

### Risk of bias

**Figure 2:**
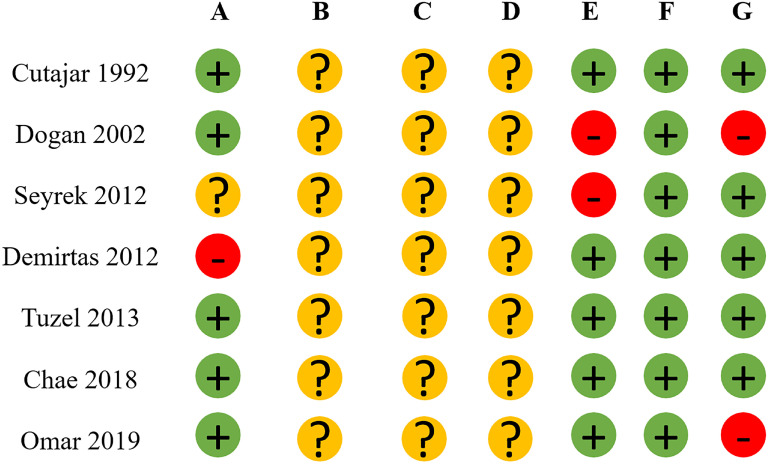
Risk of bias of randomized controlled trials.

All studies demonstrated low risk regarding reporting bias but were not clear about selection, performance, and detection bias according to RoB 2 criteria (Figure-2).

Specifically, Demirtas 2012 was deemed high risk in selection bias because "patients were divided into two groups according to prophylactic antibiotic," and Seyrek 2012 was considered unclear because "patients were randomized into two groups according to the type of antibiotic used" (13, 14).

Regarding attrition bias, Dogan 2002 and Seyrek 2012 were categorized as high risk because "urine and blood culture were taken only from febrile patients" and "after randomization, 7 patients were excluded because of purulent urine from the access needle," respectively (14, 15).

In terms of other bias, Dogan 2002 reported a "high rate of resistance to fluoroquinolones in isolated bacteria," and Omar et al. 2019 used two different antibiotics (15, 16).

### Characteristics of included studies

Cutajar 1992 conducted one of the pioneering studies comparing antimicrobial regimens in patients undergoing PCNL. In this early investigation, 70 patients undergoing the procedure were divided into two groups: one receiving a single dose of cefuroxime or norfloxacin during the induction of anesthesia and six additional doses after surgery. The study found no significant difference between the groups in terms of outcomes related to sepsis, bacteriuria, and bacteremia (17).

Dogan 2002 conducted a comparative study involving 81 patients undergoing PCNL, comparing a single dose of antibiotic during the induction of anesthesia with postoperative antibiotic prophylaxis until nephrostomy tube withdrawal. The study found no significant difference between the groups concerning bacteriuria, bacteremia, positive stone cultures, and postoperative fever. Notably, the factors associated with postoperative fever were a duration of surgery ≥ 102 minutes (p = 0.011) and the use of at least 23 L of irrigation fluid (p = 0.028) (15).

Seyrek et al. 2012 conducted a RCT involving 191 patients to assess the impact of postoperative antibiotic therapy in patients undergoing PCNL. The population was divided into two large groups based on the chosen antimicrobial (ampicillin-sulbactam and cefuroxime), and these groups were further divided into subgroups receiving single-dose prophylaxis, an additional dose 12 hours after prophylaxis, and prophylactic dose until nephrostomy tube removal. The analysis of the SIRS outcome showed no significant difference between groups (p = 0.44). The authors concluded that a single-dose administration is sufficient to prevent infectious complications (14).

Demirtas et al. 2012 conducted a study on 90 patients undergoing PCNL who were administered either ciprofloxacin or ceftriaxone. The patients were further divided into subgroups based on the drug dosage: a single dose during the induction of anesthesia, antibiotic until 12 hours after surgery, and antibiotic until nephrostomy removal. The study concluded that there was no significant difference in SIRS outcomes with respect to the use of antimicrobials (p = 0.52). Additionally, the study demonstrated no statistical association between the positivity of stone culture, renal pelvis urine culture, and postoperative urine culture in the development of SIRS in the population (13, 15).

Tuzel et al. 2013 compared 36 patients using ceftriaxone during the anesthetic induction of PCNL with 37 patients using third-generation oral cephalosporin until the removal of the nephrostomy tube. The study found no difference between the groups when evaluating outcomes such as fever (p = 0.52), positive culture of the renal pelvis (p = 0.32), stone (p = 0.47), and urine culture on the day of nephrostomy removal (p = 0.54). The conclusion drawn was that a single-dose regimen could be recommended for patients undergoing PCNL (18).

Chae et al. 2018 conducted a study involving 40 patients randomized into two groups: the first group received 2g of ceftriaxone 30 minutes before the procedure, and the second group received the same drug preoperatively plus oral cefdopoxime proxetil for three days. The study found no significant difference in postoperative fever > 38.0 ºC (p = 0.3), positive stone culture (p = 0.8), and SIRS (p = 1.0), demonstrating no superiority in extended postoperative antibiotic prophylaxis (19).

Omar et al. 2019 randomized 84 patients into two groups to evaluate a single dose of ciprofloxacin versus cefotaxime during anesthetic induction and 12 hours after the procedure. The incidence of postoperative fever was higher in the group that received cefotaxime (p = 0.002). However, there was no significant difference between the groups regarding the outcomes of length of hospital stay (p = 0.7), positive stone culture (p = 0.6), and positive urine pelvic culture (p = 0.4). The conclusion drawn was that the single-dose ciprofloxacin regimen was more effective for patients undergoing PCNL (16). Table-1 summarizes the studies included in this meta-analysis.

**Table 1:** Baseline characteristics of included randomized controlled trials

Study	Country	Design	Inclusion criteria	Definition of SIRS or Sepsis	Procedure	Patients, n
Cutajar 1992 (17)	Malta	RCT	Patients undergoing PCNL	Bacteremia was defined as the presence of bacteria in the blood (not necessarily associated with urinary tract infection) and septicemia was diagnosed when the patient developed pyrexia and rigors.	Norfloxacin before PCNL and post-operatively for a total of 6 doses vs. A single intravenous dose of cefuroxime given before PCNL	35 vs. 35
Dogan 2002 (15)	Turkey	RCT	Patients who had sterile urine preoperatively and a large stone burden or stones resistant to SWL	NA	Ofloxacin per day until removal of the nephrostomy catheter vs. A single dose of ofloxacin intravenously during induction of anesthesia	38 vs. 43
Demirtas et al. 2012 (13)	Turkey	RCT	Patients undergoing PCNL	SIRS was defined as two or more of these criteria: white blood cell count < 4,000 or >12,000, heart rate >100 per minute, fever <36°C or >38°C, respiratory rate >20 per minute. Urosepsis was defined as bacteriuria or bacteremia with SIRS positive criteria.	The first subgroup had daily dose antibiotic (ciprofloxacin or ceftriaxone) continued after the first preoperative dose antibiotic and until nephrostomy tube was extracted. The second subgroup was administered a preoperative single dose (ciprofloxacin or ceftriaxone); the postoperative was discontinued following the one given in the 12th hour. vs. A single dose of antibiotics (ciprofloxacin or ceftriaxone), rather than postoperative dose.	60 vs. 30
Seyrek et al. 2012 (14)	Turkey	RCT	Patients undergoing PCNL	SIRS was defined as two or more of these criteria: white blood cell count < 4,000 or >12,000, heart rate >100 per minute, fever <36°C or >38°C, respiratory rate >20 per minute.	Sulbactam-ampicillin 30 minutes before surgery, and then every 6 hours until removal of the nephrostomy tube; sulbactam-ampicillin 30 minutes before and 12 hours after surgery; cefuroxime 30 minutes before and 12 hours after surgery; or cefuroxime 30 minutes before surgery, and then every 8 hours until removal of the nephrostomy tube. vs. Sulbactam-ampicillin 30 minutes before surgery or cefuroxime 30 minutes before surgery	128 vs. 63
Tuzel et al. 2013 (18)	Turkey	RCT	Patients with renal stones > 2 cm and with preoperative sterile urine who underwent PCNL	NA	Ceftriaxone plus an oral third-generation cephalosporin until nephrostomy catheter withdrawal. vs. A single dose of ceftriaxone during induction of anesthesia 30 minutes before the operation	37 vs. 36
Chae et al. 2018 (19)	Korea	RCT	Patients undergoing PCNL	SIRS was defined as two or more of these criteria: white blood cell count < 4,000 or >12,000, heart rate >100 per minute, fever <36°C or >38°C, respiratory rate >20 per minute.	Ceftriaxone preoperatively and additional oral cefpodoxime proxetil for three days vs. A single dose of ceftriaxone 30 minutes before the PCNL	20 vs. 20
Omar et al. 2019 (16)	Egypt	RCT	Patients undergoing PCNL	NA	Cefotaxime divided into 2 doses, 30 minutes before induction of anesthesia and 12 hours later vs. A single dose of ciprofloxacin	43 vs. 41

SIRS = systemic inflammatory response syndrome; PCNL = Percutaneous Nephrolithotomy; SWL = Shock Wave Lithotripsy; RCT = randomized controlled trials; NA = not available.

### Outcomes

**Figure 3:**
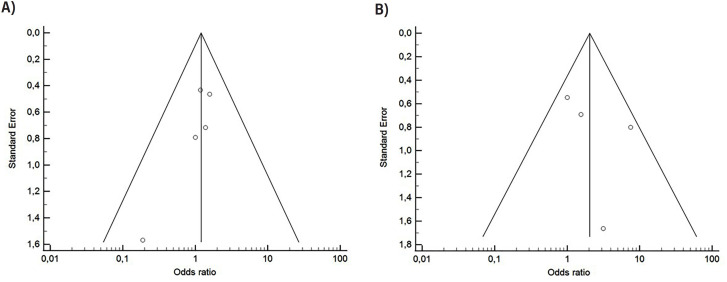
Funnel plot – (A) RCTs included in the meta-analysis for the risk of SIRS or sepsis; (B) RCTs included in the meta-analysis for the risk of fever.

**Figure 4:**
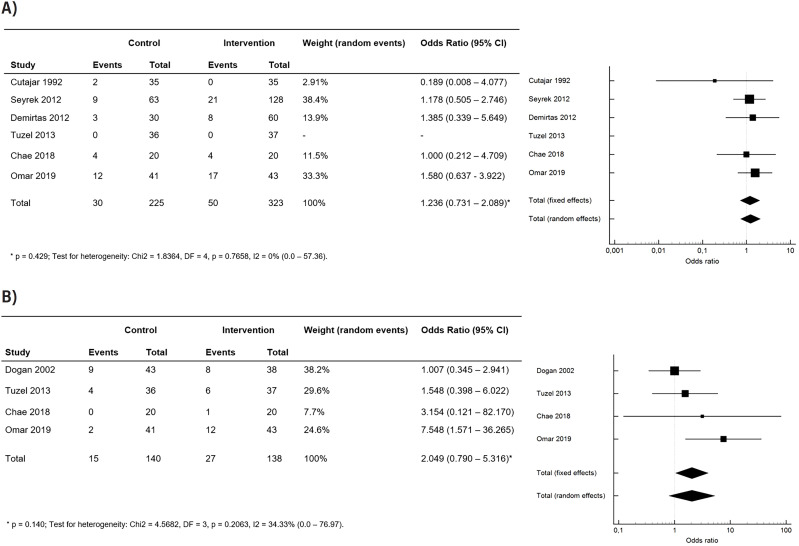
Forest plot – (A) SIRS or sepsis in control vs. intervention; (B) fever in control vs. intervention.

The outcome of SIRS/sepsis was extracted from six of the included studies, while the postoperative fever outcome was extracted from four studies. Figure-3 displays funnel plots, and Forest plots in Figure-4 illustrate that there was no statistical association between the use of postoperative antibiotic prophylaxis and the occurrence of SIRS/sepsis (OR 1.236, 95% CI 0.731 – 2.089, p=0.429) and postoperative fever (OR 2.049, 95% CI 0.790 – 5.316, p=0.140).

## DISCUSSION

This meta-analysis provides evidence suggesting that there is no reduction in the incidence of SIRS/sepsis or fever when patients receive antibiotics for an extended period beyond the induction of anesthesia in PCNL procedures. The RCTs included in this meta-analysis compared patients who received postoperative antibiotic prophylaxis until nephrostomy tube withdrawal to those who received a single dose of antibiotic during the induction of anesthesia.

In the context of PCNL, infection is a concerning complication, leading to prolonged hospital stays and posing life-threatening risks (3). Recognized risk factors for infection after PCNL include a prior positive urine culture, the presence of a nephrostomy tube or double J catheter, longer operative time, larger stones, and diabetes (2, 3, 20-22). It’s important to note that the majority of RCTs included in this meta-analysis excluded patients with a history of urinary tract infection weeks prior to PCNL and other known risk factors such as immunocompromised status and the presence of an indwelling catheter (13-19). As a result, the findings of this meta-analysis are applicable primarily to patients without some of the major risk factors for infection after PCNL.

Currently, there is a consensus among Infectious Diseases societies that there is no need to continue antibiotics for prophylactic purposes in the postoperative period, even in the presence of drains, with a level of IA evidence, as it does not reduce the incidence of infectious complications (7, 23). The indiscriminate use of antimicrobials beyond the appropriate time can lead to the selection of multidrug-resistant bacteria, creating a threatening scenario in terms of reducing therapeutic treatment options. The practice of Antimicrobial Stewardship (AMS) emerges as a necessary option in the surgical scenario, especially in Urology. AMS involves a set of practices to optimize the prescription of antibiotics when necessary, reducing patient exposure to the selection of multidrug-resistant microorganisms, improving the safety of medical care, and also reducing costs to the health service. Despite this general orientation, it is estimated that antimicrobial prescription errors occur in up to 68% of urological infections (8, 24). Therefore, more specific studies in the field of Urology are necessary to convince urologists to prescribe antibiotics according to the current best practices.

Recent meta-analyses on the topic have combined patients in different scenarios, including both pre- and postoperative use of antibiotics to prevent infection in patients undergoing PCNL. Additionally, these analyses have included different study designs, combining RCTs with retrospective studies in the same meta-analysis. This approach makes it challenging to analyze different populations within the same study (25).

In contrast, studies focusing exclusively on the preoperative scenario have indicated that seven days of oral antibiotics before PCNL can reduce the incidence of SIRS/sepsis, as well as the positivity of intraoperative urine culture and stone culture (26). It’s important to note that many, but not all, of the patients included in these studies had some infectious risk factors, such as larger stone size, positive preoperative urine culture, dilated pelvicalyceal system, or the presence of an indwelling ureteral stent or nephrostomy tube (26). However, to the best of our knowledge, this is the first meta-analysis exclusively focusing on studies that compared patients receiving postoperative antibiotic prophylaxis until nephrostomy tube withdrawal with those receiving a single dose of antibiotic during the induction of anesthesia.

This meta-analysis has enhanced the level of evidence by exclusively selecting RCTs for the study population, thereby minimizing potential biases. Additionally, the intervention population focused solely on patients receiving postoperative antibiotics, eliminating confounding factors from other perioperative periods. However, it is essential to acknowledge some limitations in this meta-analysis. Key variables, such as the choice of antibiotic, the patient’s risk for infection, and other clinical factors that may influence infectious outcomes, were not analyzed. Additionally, although none of the RCTs explicitly mentioned mini PCNL, a recent meta-analysis comparing mini PCNL to standard PCNL suggested no significant difference in infection complications between the two procedures (27). All PCNL procedures included in this meta-analysis were performed with patients in the prone position. While studies comparing prone to supine positions have demonstrated no significant difference in infection complications (28-30), this aspect should still be considered.

The meta-analysis findings underscore the importance of adopting a drug-sparing strategy, especially in the current pharmacological landscape where few new antimicrobials are anticipated. This approach becomes crucial to minimize unnecessary exposure of patients to the potential side effects of antibiotics. Furthermore, it plays a pivotal role in preventing the selection of multidrug-resistant microorganisms, for which therapeutic options are limited. In light of these findings, it is recommended that postoperative prophylactic antibiotics should not be administered to patients undergoing PCNL. This recommendation aligns with the goal of optimizing antimicrobial use, reducing the risk of antibiotic-related complications, and contributing to the broader strategy of antimicrobial stewardship.

## CONCLUSIONS

We conclude that there is no benefit regarding the use of postoperative antibiotic prophylaxis until nephrostomy tube withdrawal in patients undergoing PCNL. Antibiotic prophylaxis should be administered until induction of anesthesia of PCNL..
